# Supplementary material for: A Novel bHLH Transcription Factor PtrbHLH66 from Trifoliate Orange Positively Regulates Plant Drought Tolerance by Mediating Root Growth and ROS Scavenging
Source: Int J Mol Sci. 2022 Nov 30;23(23):15053. doi: 10.3390/ijms232315053 (PMC9740576; doi:10.3390/ijms232315053)
Supplement: Supplementary file 1 [file ijms-23-15053-s001.zip › Table S1.pdf]

Table S1. Primer sequences used for qRT-PCR analysis of drought-related genes in *PtrbHLH66* ectopic expressing Arabidopsis

Name	Accession number	Primer sequences (5'-3')	
		Forward	Reverse
AtSOD	NM_100757.4	AACTGCCACCTTCACAAT	AACAGCCCTACCAACAAT
AtPOX	NM_100404.4	CTTGCCGCTAAACTTCTA	CATCCACCACTTCATAACC
AtCAT	NM_101913.4	GAGGAGCCAATCACAGCC	AGAACCAAGCGACCAACC
AtP5CS	NM_129539.2	GCAGGCAAAGGCTTCGTT	TTCCCATCAAGTTCAGTC
AtNCED3	NM_112304.3	GGACGGAACTAAATCACC	AAATCCCGAATCTTGC
AtNCED9	NM_106486.3	TCAGAAACGGAGCAAATC	GTAAACCGACAAGCGTAG
AtZEP	NM_180954.3	GGTCAACGCTATGAAGGT	CACTACGGCCAAACAAAT
AtABA2	NM_104113.5	AGGAGCCACAGGGATAGG	TGGACTCACCACGAAGCA
AtAAO3	NM_128273.3	TGCTTATGGTCTCGGTATGG	TAACGGCTTCACAACTGCTC
AtDREB1A	AB007787.1	CGGTAAGTGGGTTTGTGAGG	CGAAATTGAGACAGGCTGAT
AtDREB2A	AB007790.1	GGCTGAGCGAGTTTGAACAT	GCAACAGATAGCGAATCCTG
AtDREB3	AB007788.1	AAAATGGGTGTCGGAGATTC	AGTCAGCGAGTTCAGGGAAG
AtRD20	NM_128898.4	AAACTTAGCGTTCAGC	TCCTTCGGTGTCATAG
AtRD29A	NM_124610.3	GTGACGACGAAGTTACCTAT	CTCTTCTTCTCCTCCAATCT
AtERD	NM_179287.2	TCTGAAGAAGCCAACACT	GAAACAAATCCATAACCC
AtACTIN	NM_001338358.1	GAAACCCTCGTAGATTGGCA	CTCTCCCGCTATGTATGTCGC
