# Supplementary material for: A Novel bHLH Transcription Factor PtrbHLH66 from Trifoliate Orange Positively Regulates Plant Drought Tolerance by Mediating Root Growth and ROS Scavenging
Source: Int J Mol Sci. 2022 Nov 30;23(23):15053. doi: 10.3390/ijms232315053 (PMC9740576; doi:10.3390/ijms232315053)
Supplement: Supplementary file 1 [file ijms-23-15053-s001.zip › Table S2.pdf]

**Table S2. Primer sequences used for qRT-PCR analysis of drought-related genes in *PtrbHLH66* homolog silencing lemon**

| Name | Accession number | Primer sequences (5'-3') | |
|---|---|---|---|
| | | Forward | Reverse |
| *ClSOD* | AF318938.1 | CCGTTTACTGGTCGTTT | TGACACCCTCAGTTCCAC |
| *ClPOX* | KT374296.1 | CCGTAGACGAGATGGTGA | CGTAAAGACGCTTGGAGA |
| *ClCAT* | KF640697.1 | CGGTATTGTGGTTCCTGG | CCCTCGTGGTGATTGTTG |
| *ClP5CS* | XM_025093600.1 | AGGAAATGCTGCCACCCA | CGGAAGAAGAAAGCGAGT |
| *ClNCED3* | AB219179.1 | CAGCAACCCAAGGAAA | TGCGTCCAAGGCTAAA |
| *ClNCED9* | AB219172.1 | AACAGCCTTACCCGAATC | AGCGAAGTTACCGCCAAT |
| *ClZEP* | AB114670.1 | GGTCAACGCTATGAAGGT | CACTACGGCCAAACAAAT |
| *ClABA2* | NM_001288867.1 | TTGGGATTCTAAGATAAAGG | GACAGCAGGCGATGAG |
| *ClAAO3* | XM_006487735.3 | AAGTTGGCACAATCCTAT | ATTCCTGTCTCATCCCTC |
| *ClDREB1A* | XM_006490258.3 | GGTCATCGCCAGAGTCACGT | GACTTCTTGTTCGGCTCCCT |
| *ClDREB2A* | XM_006478914.3 | GTCTGTCGGCAATTCATCTC | CACCCATCATCAAATCACTC |
| *ClDREB3* | XM_006421783.2 | GGGTCTCCGAAATCCGTGAG | TGCTGAGTTGCCCTTGATGC |
| *ClRD20* | XM_025102476.1 | GACTTATTCAGGGTTGCG | CTCGGTGTCGTATGTTCC |
| *ClRD29A* | XM_006421853.2 | AAAGGTGCTGTAGGTTCGTG | GTTGTTATTGTTGCGTCCTG |
| *AtERD* | XM_025097036.1 | TCCGAATCCTCCACTT | AACAGCACAGCCGTAA |
| *ClACTIN* | XM_006464503.3 | CGTATGAGCAAGGAAATCACAG | ATTGATCCTCCAATCCAAACAC |
