# Supplementary figures and images for: A Novel bHLH Transcription Factor PtrbHLH66 from Trifoliate Orange Positively Regulates Plant Drought Tolerance by Mediating Root Growth and ROS Scavenging
Source: Int J Mol Sci. 2022 Nov 30;23(23):15053. doi: 10.3390/ijms232315053 (PMC9740576; doi:10.3390/ijms232315053)

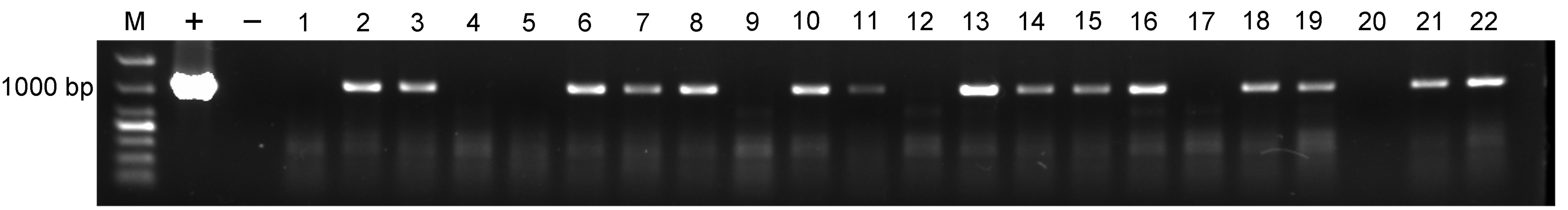

Supplement: Supplementary file 1 [file ijms-23-15053-s001.zip › Figure S1.jpg]

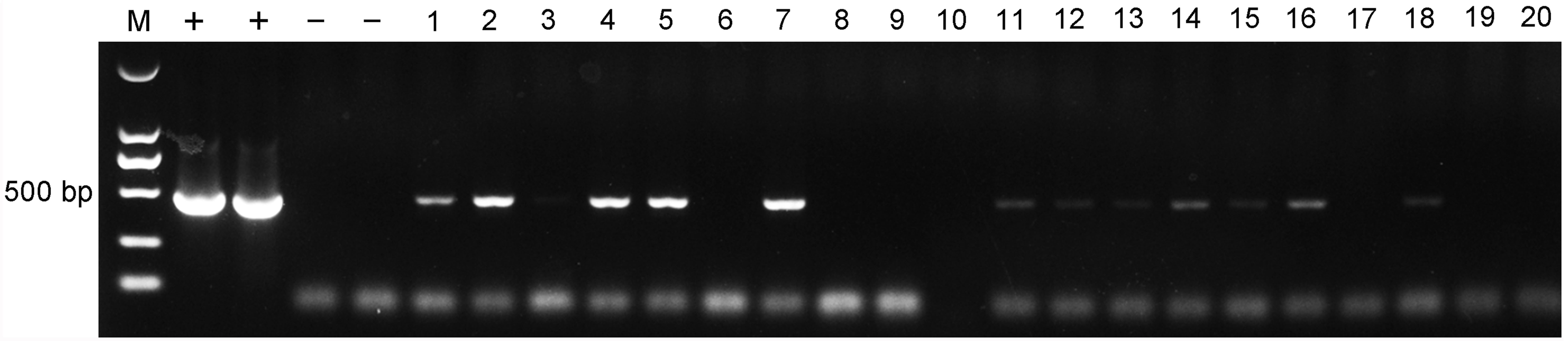

Supplement: Supplementary file 1 [file ijms-23-15053-s001.zip › Figure S2.jpg]
